# Early developmental milestones associated with tics and psychopathological comorbidity: An EMTICS study

**DOI:** 10.1007/s00787-025-02807-5

**Published:** 2025-07-17

**Authors:** Tamar Steinberg, Dana Feldman-Sadeh, Alan Apter, Yael Bronstein, Noa Elfer, Miri Carmel, Elena Michaelovsky, Abraham Weizman, Matan Nahon, Danny Horesh, Astrid Morer, Blanca Garcia Delgar, Anette Schrag, Silvana Fennig, Pieter J. Hoekstra, Andrea Dietrich, the EMTICS collaborative group, Valentina Baglioni, Juliane Ball, Noa Benaroya-Milshtein, Emese Bognar, Bianka Burger, Judith Buse, Francesco Cardona, Marta Correa Vela, Carolin Fremer, Blanca Garcia-Delgar, Julie Hagstrøm, Tammy J. Hedderly, Isobel Heyman, Chaim Huijser, Marcos Madruga-Garrido, Anna Marotta, Davide Martino, Pablo Mir, Norbert Müller, Kirsten Müller-Vahl, Alexander Münchau, Peter Nagy, Valeria Neri, Alessandra Pellico, Ángela Periañez Vasco, Kerstin J. Plessen, Cesare Porcelli, Renata Rizzo, Veit Roessner, Daphna Ruhrman, Jaana M. L. Schnel, Paola Rosaria Silvestri, Tamar Steinberg, Friederike Tagwerker Gloor, Zsanett Tarnok, Susanne Walitza, Elif Weidinger, Noa Benaroya-Milshtein

**Affiliations:** 1 Department of Child and Adolescent Psychiatry, Schneider Children’s Medical Center of Israel, 4920235 Petach Tikva, Israel; 2 School of Medicine, Faculty of Medical and Health Sciences, Tel Aviv University, Tel Aviv, Israel; 3 Felsenstein Medical Research Center, Faculty of Medical & Health Sciences, Tel Aviv University, Tel Aviv, Israel; 4 Department of Psychology, Faculty of Social Science, Bar-Ilan University, Ramat Gan, Israel; 5 Department of Child and Adolescent Psychiatry and Psychology, Institute of Neurosciences, Hospital Clinic Universitari, Barcelona, Spain; 6 Department of Clinical Neuroscience, Queen Square UCL Institute of Neurology, University College London, London, UK; 7 Department of Child and Adolescent Psychiatry, University Medical Center Groningen, University of Groningen, Groningen, The Netherlands; 8 Accare Child Study Center, Groningen, The Netherlands

**Keywords:** Developmental milestones, Tourette syndrome, Tic disorders, Attention deficit hyperactivity disorder (ADHD), Obsessive compulsive disorder (OCD)

## Abstract

**Background:**

Chronic Tic disorders (CTD) including Tourette Syndrome (TS), are associated with psychopathological comorbidities. Attention deficit/hyperactivity disorder (ADHD), autism spectrum disorder (ASD) and other comorbidities have been linked to delays in early developmental milestones. Few studies have investigated the relationship between early developmental milestones, tic severity, and related comorbidities.

**Methods:**

383 participants aged 3–16 years (76.8%, *n* = 294 boys) with CTD from the baseline assessment of the* European Multicenter Tics in Children Study* (EMTICS), were evaluated for: retrospective early developmental milestones (sitting, walking, first words, complete a sentence, bladder and bowel control), tic severity, tic-related functional impairment, obsessive–compulsive disorder (OCD), ADHD, oppositional defiant disorder (ODD) and suspected ASD. Data was collected using gold-standard self and clinician reporting instruments. Analyses included Pearson correlations and logistic regressions.

**Results:**

Correlations between the acquisition of developmental milestones and tic severity or impairment were significant with small effect sizes (severity of motor tics and tic impairment were correlated with walking (r = .11), while vocal tics were correlated with first words (r = .12)). Logistic regression revealed that delayed acquisition of first words was significantly associated with ADHD, ODD and suspected ASD (Odds Ratio (ROR): 1–1.13, 1.04–1.22, 1.05–1.21, respectively), while delayed walking acquisition was associated with OCD (ROR: 1.01–1.27).

**Discussion:**

This study highlights the association between early developmental milestones and later psychopathological comorbidities in CTD patients. These findings emphasize the need for further research to distinguish between children with only tics and those with tics and psychopathological comorbidities, to improve early detection of individuals at risk.

## Introduction

Chronic Tic Disorders (CTD), including Tourette Syndrome (TS), are neurodevelopmental conditions characterized by the persistent presence of sudden, rapid, recurrent, non-rhythmic motor movements and/or vocalizations lasting at least one year with onset before the age of 18 [1, 2]. According to studies based on clinical samples [3], TS typically manifests early in life and is frequently accompanied by high rates of psychopathological comorbid conditions throughout an individual's lifespan [3, 4]. The most common comorbidity is attention deficit/hyperactivity disorder (ADHD), followed by obsessive–compulsive disorder (OCD) and autism spectrum disorder (ASD) [5]. Other associated comorbidities include mood disorders, anxiety disorders, oppositional defiant disorders (ODD) and various disruptive, impulse-control, and conduct disorders [4–9]. 

Psychopathological comorbidities, such as ADHD and ASD have been linked to delays in early developmental milestones [10–13]. Furthermore, TS patients with psychopathological comorbidities, such as communication disorders and ADHD, may exhibit significant delays in motor and language acquisition [14]. Research suggests that TS and ADHD are linked to less efficient brain connectivity among regions, particularly showing disrupted topological organization with decreased short-range connectivity in the default mode network and frontoparietal network [15]. Additionally, shared factors such as prenatal, birth and postnatal complications may contribute to both CTD and developmental delays [16–18].

Developmental delays were also associated with other psychopathological comorbidities. For instance, children with anxiety disorders have been found to exhibit higher rates of developmental delays in areas such as speech, motor, or bladder and bowel control [19]. Similarly, parents of children with ODD have reported developmental difficulties in bladder/bowel control and language, including delays in speaking their first words [20]. The United Kingdom-based AVON longitudinal birth cohort study further reveled that children with obsessions and compulsions had significantly higher rates of soiling, indicating developmental challenges associated with OCD [21]. The Copenhagen Perinatal Cohort longitudinal study, one of the few prospective studies following individuals from birth, demonstrated that delayed acquisition of motoric milestones, including sitting and walking, was correlated with later onset of adult schizophrenia [22]. This rare prospective birth cohort design allows for more reliable documentation of the relationship between early developmental milestones and later psychopathology. These findings highlight the importance of monitoring developmental milestones during childhood to identify individuals at risk for future psychopathological comorbidities.

To our knowledge, the first investigation into developmental milestones in TS was conducted by Comings and Comings in 1987. Their study compared 247 TS patients with 47 controls and found no significant differences in the ages of first talking or walking. However, they did identify significant delays in toilet training among TS patients, including later age of first toilet training, last bed-wetting, and bowel control [23]. This area of research remained relatively neglected for several decades until Cravedi et al. (2018) examined developmental trajectories in 174 children and adolescents with TS. Their study demonstrated that developmental delays are more common in individuals with both TS and neurodevelopmental comorbidities, such as ADHD, compared to those with TS alone [15, 24]

Given the limited research, the connection between developmental delays and later-life tic severity and psychopathological comorbidities remains inconclusive. To address this gap, we retrospectively examined early developmental milestones in a large sample of youth (ages 3–16) with chronic tics from the European Multicenter Tics in Children Study (EMTICS) [25]. Our study aimed to investigate the developmental milestones of individuals with chronic CTDs. Specifically, we sought to explore the relationship between the age of acquiring early developmental milestones (motor, language, and toilet training) and both tic severity and impairment, as well as psychopathological comorbidities, including OCD, ADHD, ODD, and suspected ASD. We hypothesized that delayed acquisition of these milestones would predict greater tic severity and impairment, along with more severe comorbid symptoms or higher rates of comorbid psychopathologies.

## Methods

### Participants and procedure

The sample included 383 children and adolescents aged 3 to 16 years with Tourette syndrome (TS) or chronic tic disorder (CTD) who participated in the baseline assessment of the EMTICS study, which investigates the role of genetic, autoimmune, and psychosocial factors in the onset and progression of tics [24]. Participants were recruited from sixteen child mental health or pediatric neurology outpatient clinics across Europe and Israel, as well as through advertisements directed at patient organizations and health professionals. All participants who met the diagnostic criteria for TS or CTD according to the DSM-IV-TR (American Psychiatric Association) were invited, along with their parents or guardians, to participate in the study [1].

Inclusion criteria required participants to be between 3 and 16 years old, with a confirmed diagnosis of TS or CTD based on DSM-IV-TR criteria [1]. Additionally, for the current study, only participants whose parents had completed all questions related to early developmental milestones were included. Exclusion criteria included severe medical or neurological illness, recent antibiotic treatment within the past month, or inability to comply with study procedures. Written informed consent was obtained from parents or legal guardians, and participating adolescents provided either written assent or consent, depending on local medical-ethical regulations. Ethical approval was granted by the relevant Research Ethics Committees at each participating center.

Baseline measures, including parent-reported questionnaires and a clinical interview, were collected during the initial EMTICS visit. Additionally, a clinician confirmed the diagnosis of TS or CTD and assessed for ADHD and OCD according to DSM-IV-TR criteria [1].

### Measures

Demographic information, including the child's sex, age at evaluation, study site, and ethnicity, was collected through a demographic questionnaire.

Early Developmental Milestones were gathered using the Modified Schedule for Risk and Protective Factors in Early Development (MSRPFED) [24]. Parents reported the age (in months) at which their child acquired eight developmental milestones from early life: sitting, walking, first words, sentence completion, daytime and nighttime bladder control, and bowel control. This information created eight continuous developmental measures for analysis. Developmental delays were assessed using the Denver II assessment for motor and language development [25], with suspected delays determined using the 90th percentile age norms. Toilet training and delays differentiated by sex were assessed based on the 75th percentile, following the methodology of Schum et al. [26] For the developmental milestones norms, refer to Appendix B, Table 6. Consequently, an additional dichotomous variable was created for each developmental milestone to identify children with developmental delays.

Tic severity and impairment symptoms were measured using the clinician-rated *Yale Global Tic Severity Scale* (YGTSS) [27, 28]. Each of the following dimensions was rated on a five-point scale: number, frequency, intensity, interference, and complexity over the past week. Scores were calculated for motor tic severity (range: 0–25), vocal tic severity (range: 0–25), total tic severity (range: 0–50), and the tic impairment score (subjective evaluation of impairment caused by tics, range: 0–50). In the current study, Cronbach’s alpha values were calculated as follows: α = 0.88 for the total tic severity scale, α = 0.82 for the motor subscale, and α = 0.89 for the vocal subscale. Total tic severity cut-offs were defined as follows: minimal tics < 10, mild tics: 10–20, moderate tics: 20–40, and severe tics: 40–50, based on the work of Leckman and colleagues [27].

Obsessive–Compulsive Disorder (OCD) symptoms were assessed using the clinician-rated Children's Yale-Brown Obsessive–Compulsive Scale (CY-BOCS) [32], which evaluates five dimensions: time, interference, distress, resistance, and control associated with obsessions and compulsions over the past week. Each dimension is rated on a five-point scale. The scale provides three summary scores: obsession severity (ranging from 0 to 20; α = 0.92), compulsion severity (ranging from 0 to 20; α = 0.93), and total OCD severity (ranging from 0 to 40; α = 0.94). A dichotomous diagnosis of OCD was based on DSM-IV-TR criteria, as determined by a trained clinician, or a CY-BOCS total severity score of 16 or higher, following the cut-off established by Scahill et al. [29].

Attention Deficit/Hyperactivity Disorder (ADHD) symptoms were assessed using the abbreviated version of *the Swanson, Nolan, and Pelham, version IV (SNAP-IV)* rating scale, which evaluates symptoms over the past week on a four-point scale. This scale yields three scores: inattention score (9 items, range 0–27; current study α = 0.91), hyperactivity-impulsivity score (9 items, range 0–27; α = 0.90), and combined score (18 items, range 0–54; current study α = 0.94), with higher scores indicating greater symptom severity [30]. A dichotomous diagnosis of ADHD was determined based on DSM-IV-TR criteria, as diagnosed by a trained clinician, and included any of the ADHD subtypes (inattentive, hyperactive, or combined) [31].

Oppositional Defiant Disorder (ODD) symptoms were assessed using the parent-rated* Swanson, Nolan, and Pelham, version IV* (SNAP-IV) abbreviated version rating scale. ODD symptoms were evaluated over the past week on a four-point scale, with higher scores indicating greater symptom severity (8 items, range 0–24; current study α = 0.91). A dichotomous ODD variable was defined as scoring above the 95th percentile cut-off on the SNAP-IV questionnaire (ODD mean score ≥ 1.88) [31].

Suspected Autism Spectrum Disorder (ASD) symptoms were assessed using the parent-rated Autism Spectrum Screening Questionnaire (ASSQ) [32]. A three-point scale was employed to evaluate symptoms across four domains: social interaction, communication, restricted and repetitive behaviors, and motor clumsiness, along with other associated symptoms. Higher scores indicated greater suspected ASD symptom severity (27 items, range 0–54; α = 0.90). A dichotomous variable for suspected ASD was defined as scoring above the established cut-off (ASSQ score ≥ 19) [32].

## Statistical analysis

Missing data patterns were examined using Little's MCAR (Missing Completely at Random) test, incorporating variables such as developmental milestones, tics, OCD, ADHD, ODD, suspected ASD, sex, age at visit, ethnicity, and site.

Correlation matrices were constructed to evaluate both intra-correlations and inter-correlations among early developmental milestones, tic severity and impairment, and the severity of psychopathological comorbidity symptoms (all continuous variables).

Due to the weak correlations with continuous variables, we proceeded with our analysis using dichotomous dependent variables for ADHD, OCD, and ODD (based on DSM-IV-TR criteria or established cut-offs from gold standard report measures).

To evaluate whether developmental milestones are related to the presence of psychopathological comorbid diagnoses, logistic regression analyses were performed separately for each diagnosis, with the presence of ADHD, ODD, OCD, and suspected ASD as dependent variables (0 = without the respective comorbidity; 1 = with the respective comorbidity). Independent variables included the age in months for each developmental milestone (sitting, walking, first words, sentence completion, daytime/nighttime bladder control, and bowel control). Covariates such as sex, age, and site were also included.

Results were reported using odds ratios (OR), where OR values below, equal to, or above 1 reflect lower, neutral, or higher odds of the outcome, respectively. Model accuracy was evaluated using a classification table to compare observed and predicted outcomes. All analyses were conducted using IBM SPSS, Version 20.0 [34], with a significance level of α = 0.05.

## Results

### Sample characteristics

The initial sample consisted of 709 participants. Only participants whose parents had completed all questions related to early developmental milestones were included. Missing data were assessed using Little's MCAR test (considering variables such as sex, age at visit, ethnicity, study site, tics, OCD, ADHD, ODD, and suspected ASD), which indicated that the missing data were completely random (χ^2^(523) = 564.26, *p* = 0.10). To validate this result, differences in outcome variables between participants with complete data and those who were excluded were examined using t-tests. Although significant differences were found for nighttime bowel control and ADHD (with higher scores for excluded participants), the effect sizes were relatively small (Cohen's d ≤ 0.2). The final sample included 383 participants.

The majority of participants (87.5%, *n* = 335) were diagnosed with Tourette syndrome (TS), 11.5% (*n* = 44) with chronic motor tic disorder, and less than 1% (*n* = 1; 0.3%) with chronic vocal tic disorder. In terms of tic severity, 47% (*n* = 180) of participants exhibited moderate tic severity, 36% (*n* = 138) had mild tics, 16.2% (*n* = 62) had minimal tics, and 0.8% (*n* = 3) had severe tics. Regarding comorbidities, 31% (*n* = 121) of participants met criteria for OCD, 25.1% (*n* = 96) for ADHD, 12.8% (*n* = 49) of participants met criteria for ODD severity score and 18.8% (*n* = 72) met criteria for suspected ASD. Descriptive statistics for demographics, tic severity, impairment, and comorbid psychopathology symptoms are presented in Table 1. For intra-correlations between tics and comorbid psychopathology, refer to Appendix B, Table 7.

**Table 1:** Demographics and clinical characteristics (*n* = 383)

	N(%)	M ± SD	Range
Age		10.67 ± 2.69	3.35–16.79
Sex (male)	*294 (76.8)*		-
Ethnicity (Caucasian)	*362 (94.5)*		-
Chronic tic disorder			
Motor tic severity score^*a*^		12.42 ± 4.55	0–23
Vocal tic severity score^*a*^		7.40 ± 5.55	0–22
Total tic severity score^*a*^		19.81 ± 8.70	0–44
Tic impairment score^*a*^		14.73 ± 11.68	0–50
OCD^*b*^	*121 (31)*		
Obsessions severity score^*b*^		3.20 ± 4.69	0–17
Compulsions severity score^*b*^		3.85 ± 4.99	0–20
Total OCD severity score^*b*^		7.04 ± 8.85	0–34
ADHD	*96 (25.1)*		
Inattention severity score^*c*^		10.59 ± 6.91	0–27
Hyperactivity severity score^*c*^		8.87 ± 6.76	0–27
Combined severity score^*c*^		19.46 ± 12.58	0–54
ODD^*c*^ ODD severity score	*49 (12.8)*	8.23 ± 5.96	0–24
Suspected ASD^*d*^	*72(18.8)*	11.06 ± 8.93	0–46

*OCD*, Obsessive Compulsive Disorder; *ADHD*, Attention Deficit Hyperactive Disorder; *ODD*, Oppositional Defiant Disorder; *suspected ASD*, suspected Autism Spectrum Disorder;

^a^by YGTSS – Yale Global Tic Severity Scale; ^b^by CYBOCS – Children’s Yale-Brown Obsessive Compulsive Scale; ^c^by SNAP – Swanson, Nolan and Pelham; ^d^ by ASSQ – Autism Spectrum Screening Questionnaire

### Early developmental milestones

Table 2 presents the average age at which participants achieved early developmental milestones, as well as the percentage of participants with suspected delays. Daytime and nighttime bowel control were highly correlated (r = 0.83, *p* < 0.01) and were therefore combined into a single variable for further analysis based on clinical considerations. Table 3 shows intra-correlations between developmental milestones, with significant positive correlations within each domain (motor, language, and toilet training; r = 0.39–0.67, *p* < 0.01). Cross-domain correlations ranged from minor to moderate positive (r = 0.02–0.46, *p* = n.s.– < 0.01).

**Table 2:** Age of acquiring early developmental milestones (*n* = 383)

Developmental milestones	Md (months)	M ± SD (months)	Range (months)	Suspected developmental delay % (n)
Motor development				
Sitting	6.00	6.84 ± 1.54	3–12	2.9 (11)^a^
Walking	12.50	13.01 ± 2.48	8.5–36	8.1 (31)^a^
Language development				
First words	12.00	13.28 ± 5.98	4–52	19.8 (76)^a^
Complete a sentence	22.00	21.95 ± 7.58	8–60	18.3 (70)^a^
Toilet training				
Day bladder control	30.00	30.80 ± 13.87	10–168	8.9 (34)^b^
Night bladder control	30.00	37.34 ± 21.70	12–192	19.1 (73)^b^
Bowel control	30.00	31.85 ± 10.72	12–134	15.4 (59)^b^

^a^According to The Denver II Developmental Screening Test (1992) [25]

^b^According to Schum, et al. (2002) [26]

**Table 3:** Correlation matrix of intra-correlations between early developmental milestones (*n* = 383)

Domain	Developmental milestones	Walking	First words	Complete a sentence	Day bladder control	Night bladder control	Bowel control
Motor	Sitting	0.40**	0.22**	0.06	0.08	0.08	0.16**
	Walking	–	0.46**	0.28**	0.09	0.06	0.14**
Language	First words		–	0.68**	0.12*	0.09	0.25**
	Complete a sentence			–	0.09	0.02	0.22**
Toilet training	Day bladder control				–	0.67**	0.59**
	Night bladder control					–	0.46**
	Bowel control						–

*p < 0.05, **p < 0.01

For the developmental milestones norms, refer to Appendix B, Table 6

### Correlations between early acquisition of developmental milestones, tics, and comorbid symptoms

A Pearson correlation analysis (Table 4) revealed weak correlations overall, with small effect sizes (r < 0.21). Notably, the acquisition of sitting was not associated with any comorbid psychopathological symptoms. Motor tics and tic impairment were correlated with walking (r = 0.11–0.12), while vocal tics were correlated with first words (r = 0.12). OCD showed correlations with walking, first words, and sentence completion (r = 0.12–0.18). ADHD was correlated with walking, first words, sentence completion, and bowel control (r = 0.12–0.21). ODD was correlated with first words (r = 0.15), and suspected ASD was correlated with bladder and bowel control (r = 0.10–0.17).

**Table 4:** Correlation matrix between early developmental milestones and severity of tics and comorbid psychopathology symptoms (*n* = 383)

	Age of acquiring early developmental milestones (months)						
	Sitting	Walking	First words	Complete a sentence	Day bladder control	Night bladder control	Bowel control
Tics^a^							
Motor tic severity	−0.04	0.11*	0.02	0.05	0.04	−0.003	0.01
Vocal tic severity	−0.09	0.06	0.12*	0.08	0.05	−0.007	0.08
Total tic severity	−0.08	0.09	0.08	0.07	0.06	−0.004	0.06
Impairment	−0.04	0.12*	0.08	0.07	0.07	0.03	0.09
OCD severity^b^							
Obsessions	0.01	0.16**	0.15**	0.12**	0.04	−0.05	0.05
Compulsions	0.005	0.17**	0.07	0.08	0.03	−0.02	0.05
Total OCD severity	0.0*1*	0.18**	0.12*	0.11*	0.03	−0.04	0.05
ADHD severity^c^							
Inattention	0.09	0.18**	0.18**	0.13*	0.07	0.06	0.1*
Hyperactivity	0.06	0.16**	0.21**	0.12*	0.04	0.06	0.11*
Combined	0.08	0.19**	0.21**	0.13**	0.06	0.06	0.12*
ODD severity^c^	−0.05	0.08	0.15**	0.09	−0.008	−0.01	0.02
ASD severity^d^	0.08	0.05	0.1	0.03	0.17**	0.17**	0.10*

*p < 0.05, **p < 0.01

*OCD*, Obsessive Compulsive Disorder; *ADHD*, Attention Deficit Hyperactive Disorder; *suspected ASD*, suspected Autism Spectrum Disorder; *ODD*, Oppositional Defiant Disorder.

^a^by YGTSS – Yale Global Tic Severity Scale; ^b^by CYBOCS – Children’s Yale-Brown Obsessive Compulsive Scale; ^c^by SNAP – Swanson, Nolan and Pelham; ^d^ by ASSQ – Autism Spectrum Screening Questionnaire.

### Prediction of psychopathological comorbidity diagnoses by early developmental milestones

Logistic regression analyses were conducted to explore the relationship between early developmental milestones and comorbid psychopathological diagnoses (Table 5.), with the results detailed below. Unless otherwise noted, the covariates (sex, age, and site) were not significant.

**Table 5.** Logistic Regressions for predicting comorbid psychopathology diagnoses by early developmental milestones

Prediction of OCD by:	B	SE	Wald	p	Exp(B) (95% C.I)	NagelkerkeR^2^
Overall						0.122
Sitting	−0.08	0.08	0.94	0.33	0.92 (0.78–1.09)	
Walking	0.12	0.06	4.24	0.04	1.13 (1.01–1.27)	
First words	0.04	0.03	1.94	0.16	1.04 (0.98–1.10)	
Complete sentence	0.00	0.02	0.00	1.00	1.00 (0.96–1.04)	
Day bladder control	0.02	0.01	2.37	0.12	1.02 (0.99–1.05)	
Night bladder control	−0.01	0.01	2.80	0.09	0.99 (0.97–1.00)	
Bowel control	0.01	0.01	0.28	0.60	1.01 (0.98–1.03)	
**Prediction of ADHD by**:	**B**	**SE**	**Wald**	**p**	**Exp(B) (95% C.I)**	NagelkerkeR^2^
Overall						0.086
Sitting	−0.03	0.09	0.16	0.69	0.97 (0.82–1.14)	
Walking	0.12	0.06	3.66	0.06	1.12 (1.00–1.27)	
First words	0.06	0.03	3.83	0.05	1.06 (1.00–1.13)	
Complete sentence	−0.05	0.02	4.01	0.05	0.95 (0.91–1.00)	
Day bladder control	−0.03	0.03	0.97	0.32	0.98 (0.93–1.03)	
Night bladder control	0.00	0.01	0.19	0.67	1.00 (0.98–1.01)	
Bowel control	0.05	0.02	3.52	0.06	1.05 (0.98–1.10)	
**Prediction of ODD by:**	B	SE	Wald	p	Exp(B) (95% C.I)	NagelkerkeR^2^
Overall						
Sitting	−0.36	0.13	7.53	0.01	0.70 (0.54–0.90)	0.119
Walking	0.07	0.07	1.03	0.31	1.07 (0.94–1.23)	
First words	0.12	0.04	8.74	0.00	1.12 (1.04–1.22)	
Complete sentence	−0.06	0.03	3.30	0.07	0.94 (0.88–1.00)	
Day bladder control	−0.04	0.05	0.68	0.41	0.96 (0.88–1.05)	
Night bladder control	0.00	0.01	0.19	0.66	1.00 (0.98–1.02)	
Bowel control	0.02	0.04	0.16	0.68	0.1.02 (0.94–1.09)	
**Prediction of suspected ASD by:**	B	SE	Wald	p	Exp(B) (95% C.I)	NagelkerkeR^2^
Overall						
Sitting	0.086	0.09	0.22	0.64	1.05 (0.87–1.26)	0.138
Walking	−0.09	0.07	1.99	0.16	0.91 (0.80–1.04)	
First words	0.12	0.04	11.34	0.00	1.13 (1.05–1.21)	
Complete sentence	−0.06	0.03	3.75	0.05	0.95 (0.89–1.00)	
Day bladder control	0.01	0.01	0.33	0.57	1.01 (0.98–1.04)	
Night bladder control	0.01	0.01	2.09	0.15	1.01 (1.00–1.03)	
Bowel control	−0.01	0.02	0.70	0.40	0.99 (0.96–1.02)	

the models covaried sex site and age (significant results are presented in the main text)

*ADHD*, Attention Deficit Hyperactive Disorder; *OCD*, Obsessive Compulsive Disorder; *suspected ASD*, suspected Autism Spectrum Disorder; *ODD*, Oppositional Defiant Disorder.

#### OCD

The model was significant (χ^2^(10) = 34.2, *p* < 0.01), accounting for 12.2% of the variance in OCD and correctly classifying 68.7% of cases. Later acquisition of walking (OR = 1.13; 1.01–1.27, *p* < 0.05) and the child's older age (covariate) (OR = 1.16; 1.06–1.26, *p* < 0.01) increased the likelihood of an OCD diagnosis.

#### ADHD

The model predicting ADHD was also significant (χ^2^(10) = 22.97, *p* < 0.01), accounting for 8.6% of the variance and correctly classifying 75% of cases. Later acquisition of first words increased the likelihood of an ADHD diagnosis (OR = 1.06; 1.00–1.30, *p* < 0.05), while later acquisition of sentence completion reduced the likelihood (OR = 0.95; 0.91–0.99, *p* < 0.05).

#### ODD

Significant results were found (χ^2^(10) = 25.11, *p* < 0.05), contributing 11.9% to the variance in ODD and correctly classifying 87.2% of cases. Later acquisition of first words increased the likelihood of ODD (OR = 1.13; 1.04–1.22, *p* < 0.01), while later acquisition of sitting reduced the likelihood (OR = 0.70; 0.54–0.90, *p* < 0.01). Being male (covariate) also increased the likelihood of an ODD diagnosis (OR = 0.36; 0.14–0.90, *p* < 0.03).

#### Suspected ASD

The model was significant (χ^2^(10) = 34.32, *p* < 0.01), accounting for 13.8% of the variance in suspected ASD and correctly classifying 81.2% of cases. Later acquisition of first words increased the likelihood of suspected ASD (OR = 1.13; 1.05–1.21, *p* < 0.01), while sentence completion reduced the likelihood (OR = 0.95; 0.89–1.00, *p* < 0.05).

## Discussion

The current study included a large sample of children and adolescents aged 3–16 years with chronic tic disorders (CTD), aimed to explore the relationship between delayed acquisition of key developmental milestones (motor, language, and toilet training) and tic severity, tic-related impairment as well as psychopathological comorbidities. The primary finding was that delayed language acquisition, particularly speaking first words in infancy, predicted later psychopathological comorbidities associated with CTD, including OCD, ADHD, ODD and suspected ASD.

In line with our hypothesis, tic severity and impairment were correlated with developmental milestones albeit with small effect size. Interestingly, motor tics and tic impairment were associated with the age of first walking, while vocal tics were linked to delayed acquisition of first words. Similarly, Cravedi et al., (2018), identified developmental delays in children with tics and neurodevelopmental comorbidities, however, their study categorized TS individuals into clusters rather than using continuous measures [14]. In contrast, Coming and Comings (1987), in a different study design, found no significant delays in talking or walking among TS patients compared to controls, but reported significant delays in bladder and bowel control [23]. These methodological differences may explain the variance in findings between studies. Thus, further research is needed to draw definitive conclusions.

Regarding related psychopathological comorbidities, our findings revealed a correlation between OCD, both severity and diagnosis, and delays in motor (walking) and language development. This aligns with previous research indicating that children with anxiety, a common comorbidity of OCD, often experience developmental delays in motor, language, and toilet training milestones [19, 33, 34] Importantly, this study focused on tic-related OCD, and future research should further explore developmental delays in cases of non-tic-related OCD [35, 36].

Our findings corroborate previous research that links neurodevelopmental disorders to developmental delays [13, 37, 38]. Specifically, we found that delayed language acquisition and bowel control were both associated with the severity of ADHD and suspected ASD symptoms. This aligns with LeBeau et al. (2022), who reported that bowel control difficulties were linked to the severity of ADHD and ASD symptoms, while delayed speech acquisition predicted later ASD diagnoses [10]. Similarly, Gurevitz et al. (2012) identified language delay as a significant early predictor of ADHD [39, 40].

Regarding ODD, our study found a significant correlation between delayed acquisition of first words and later ODD diagnosis and severity of symptoms. This aligns with previous research showing that children with ODD often experience slower language development and less speech clarity [21]. Nevertheless more studies on ODD with or without TS are needed.

Interestingly, negative correlations were found between ADHD and suspected ASD diagnoses with sentence completion, as well as between ODD diagnoses and sitting. One possible explanation is that children with psychopathological comorbidities often exhibit highly variable developmental trajectories, resulting in uneven progress across different developmental domains. For example, LeBeau et al. (2022) identified a similar pattern, particularly noting negative correlations between ADHD and delayed language acquisition [10]. Another explanation may lie in the tendency of parents to more easily recall prominent milestones, such as first words or walking, compared to less noticeable ones like sentence completion or sitting. This highlights the potential influence of memory bias in parental reporting of developmental milestones.

This study highlights the association between early developmental delays, including language, motor acquisition, and bowel/bladder control, and the later development of psychopathological comorbidities (OCD, ADHD, ODD, and suspected ASD) in CTD patients. Identifying these relationships could improve early detection of at-risk individuals and facilitate timely, tailored interventions, potentially improving long-term outcomes and quality of life for CTD patients with comorbid psychopathologies. Furthermore, our findings underscore the importance of distinguishing between patients with tics alone and those with both tics and comorbid psychopathologies, as demonstrated in previous studies [3, 40, 41].

### Strengths and limitations

This study offers several notable strengths, such as its large sample size and thorough assessment of developmental milestones across various domains. However, there are important limitations. Participant recruitment from clinical settings could introduce selection bias, leading to a higher proportion of individuals with psychopathological comorbidities compared to the general population. Additionally, the reduction in sample size due to missing developmental milestone data should be considered, though the analysis suggests this data was missing randomly. Methodologically, the study faced constraints, such as relying solely on retrospective parental reports without supplementary measurement tools, and the absence of inter-rater reliability measures for evaluating developmental milestones, which could affect data consistency. Moreover, our study did not include systematic assessment of learning disabilities and cognitive difficulties, which could be relevant to developmental milestone acquisition, particularly language development. Given the participants'mean age, recall bias may have influenced the results; however, the large sample size may reduce individual inaccuracies. We used widely accepted developmental norms from validated tools, acknowledging the challenges in establishing universal norms. Finally, the impact of multiple comparisons on the results should be noted, though the consistency of patterns, especially regarding language delays and their link to symptom severity, supports clinical knowledge that early developmental delays signal a higher risk for multiple diagnoses later in childhood and adolescence. Future research should incorporate a prospective design, comparing healthy controls, individuals with tics only, and those with specific psychiatric disorders without tics, to better differentiate the contributions of developmental trajectories to various clinical presentations and improve the precision of associations between developmental milestones and psychopathological comorbidities.

## Data Availability

No datasets were generated or analysed during the current study.

## Appendix A

**EMTICS group authorship**: Alan Apter^1^, Valentina Baglioni^2^, Juliane Ball^3^, Noa Benaroya-Milshtein^1^ Emese Bognar^4^, Bianka Burger^5^, Judith Buse^6^, Francesco Cardona^2^, Marta Correa Vela^7^, Andrea Dietrich^8,9^, Carolin Fremer^10^, Blanca Garcia-Delgar^11^, Julie Hagstrøm^12^, Tammy J. Hedderly^13^, Isobel Heyman^14^, Pieter J. Hoekstra^8,9^, Chaim Huijser^15,16^, Marcos Madruga-Garrido^17^, Anna Marotta^18^, Davide Martino^19^, Pablo Mir^7,20,21^, Astrid Morer^11,22,23^, Norbert Müller^5^, Kirsten Müller-Vahl^10^, Alexander Münchau^24^, Peter Nagy^4^, Valeria Neri^2^, Alessandra Pellico^25^, Ángela Periañez Vasco^7^, Kerstin J. Plessen^12,26^, Cesare Porcelli^18^, Renata Rizzo^23^, Veit Roessner^6^, Daphna Ruhrman^1^, Jaana M.L. Schnell^5^, Anette Schrag^27^, Paola Rosaria Silvestri^2^, Tamar Steinberg^1^, Friederike Tagwerker Gloor^3^, Zsanett Tarnok^4,28^, Susanne Walitza^3^, Elif Weidinger^5^

^1^Child and Adolescent Psychiatry Department, Schneider Children's Medical Center of Israel, affiliated to Sackler Faculty of Medicine, Tel Aviv University, Petah-Tikva, Israel

^2^University La Sapienza of Rome, Department of Human Neurosciences, Rome, Italy

^3^Department of Child and Adolescent Psychiatry and Psychotherapy, University of Zurich, Zurich, Switzerland

4Vadaskert Child and Adolescent Psychiatric Hospital, Budapest, Hungary

^5^Department of Psychiatry and Psychotherapy, University Hospital, LMU Munich, Munich, Germany.

^6^Clinical Child and Adolescent Psychology, Institute of Clinical Psychology and Psychotherapy, TU Dresden, Germany

^7^Unidad de Trastornos del Movimiento. Instituto de Biomedicina de Sevilla (IBiS). Hospital Universitario Virgen del Rocío/CSIC/Universidad de Sevilla. Seville, Spain.

^8^Accare Child Study Center, Groningen, The Netherlands

^9^University of Groningen, University Medical Center Groningen, Department of Child and Adolescent Psychiatry, Groningen, The Netherlands

^10^Clinic of Psychiatry, Socialpsychiatry and Psychotherapy, Hannover Medical School, Hannover, Germany

^11^Department of Child and Adolescent Psychiatry and Psychology, Institute of Neurosciences, Hospital Clinic Universitari, Barcelona, Spain

^12^Child and Adolescent Mental Health Center, Mental Health Services, Capital Region of Denmark, Denmark

^13^Evelina London Children’s Hospital GSTT, Kings Health Partners AHSC, London, UK.

^14^Great Ormond Street Hospital for Children, and UCL Institute of Child Health, London, UK

^15^Levvel, Academic Center for Child and Adolescent Psychiatry, Amsterdam, The Netherlands

^16^Amsterdam UMC, Department of Child and Adolescent Psychiatry, Amsterdam, The Netherlands

^17^Neuropediatrics, Centro de Pediatría de Sevilla, Hospital Viamed Santa Ángela De la Cruz, Seville, Spain

^18^Azienda Sanitaria Locale di Bari, Mental Health Department, Child and Adolescent Service of Bari Metropolitan Area, Bari, Italy

^19^Department of Clinical Neurosciences, University of Calgary, Calgary, Canada

^20^Centro de Investigación Biomédica en Red sobre Enfermedades Neurodegenerativas (CIBERNED), Madrid, Spain

^21^Departamento de Medicina, Facultad de Medicina, Universidad de Sevilla, Sevilla, Spain

^22^Institut d'Investigacions Biomediques August Pi i Sunyer (IDIBAPS), Barcelona, Spain

^23^Centro de Investigacion en Red de Salud Mental (CIBERSAM), Instituto Carlos III, Spain

^24^Institute of Systems Motor Science, Center of Brain, Behavior and Metabolism, University of Lübeck, Lübeck, Germany

^25^Child Neuropsychiatry Section, Department of Clinical and Experimental Medicine, School of Medicine, Catania University, Catania, Italy

^26^Division of Child and Adolescent Psychiatry, Department of Psychiatry, University Medical Center, University of Lausanne, Lausanne, Switzerland

^27^Department of Clinical Neurosciene, UCL Institute of Neurology, University College London, London, UK

^28^Semmelweis University, Budapest, Hungary

### Further Acknowledgement

This research was supported by Stiftung Immunität und Seele (Burger, Müller, Schnell); and National Institute for Health Research Biomedical Research Centre at Great Ormond Street Hospital for Children NHS Foundation Trust and University College London (Heymanm, schrag); and Deutsche Forschungsgemeinschaft (DFG): projects 1692/3–1, 4–1 and FOR 2698 (Münchau); Deutsche Forschungsgemeinschaft (DFG): FOR 2698 (Roessner). We thank all colleagues at the various study centers who contributed to data collection:, Judy Grejsen (Paediatric Department, Herlev University Hospital, Herlev, Denmark); Julie E. Bruun, Christine L. Ommundsen, Mette Rubæk (Capital Region Psychiatry, Copenhagen, Denmark); Stephanie Enghardt, Benjamin Bodmer (TU Dresden, Germany); Carolin Fremer (MHH Hannover, Germany); Jenny Schmalfeld (Lübeck University, Germany); Martin L. Woods, Victoria L. Turner (Evelina London Children’s Hospital, United Kingdom); Franciska Gergye, Margit Kovacs, Reka Vidomusz (Vadaskert Hospital, Budapest, Hungary); Chen Regev, Tomer Simcha, Adi Moka, Julia Katz (Tel Aviv, Petah-Tikva, Israel); Annelieke Hagen (Levvel, Amsterdam, Netherlands); Marieke Messchendorp, Thaïra J.C. Openneer, Anne-Marie Stolte, Frank Visscher and the Stichting Gilles de la Tourette (University Medical Center Groningen, Netherlands); Maria Teresa Cáceres, Fátima Carrillo, Laura Vargas (Seville, Spain); Maria Cristina Ferro, Mariangela Gulisano (University of Catania, Italy), and all who may not have been mentioned.

### Contributors

N. Benaroya-Milshtein, T. Steinberg, D. Feldman-Sadeh, A. Apter, conceptualized and designed the study; N. Benaroya-Milshtein, T. Steinberg, M. Carmel, E. Michaelovsky, M. Nahon, P.J. Hoekstra, A. Dietrich and the EMTICS collaborative group—collected data; N. Benaroya-Milshtein, D. Feldman-Sadeh, T. Steinberg, Y. Bronstein, N. Elfer, P.J. Hoekstra and A. Dietrich were responsible for the statistical analyses; N. Benaroya-Milshtein, T. Steinberg, D. Feldman-Sadeh, P.J. Hoekstra, A. Dietrich and the EMTICS collaborative group drafted the initial manuscript; N. Benaroya-Milshtein, T. Steinberg, D. Feldman-Sadeh, A. Apter, Y. Bronstein, N. Elfer, M. Carmel, E. Michaelovsky, A. Weizman, M. Nahon, D. Horesh, A. Morer, B. Garcia Delgar, P.J. Hoekstra and A. Dietrich – critically reviewed the manuscript for important intellectual content and revised the manuscript; All authors approved the final manuscript as submitted and agree to be accountable for all aspects of the work.

## Appendix B

Table. 6

**Table 6:** Developmental Milestone Norms

Developmental Milestone	Norms of Age (in month) of Acquiring Milestone*	
**Motor development**	50th percentile	90th percentile
Sitting	8.5	10
Walking	12.5	16.5
**Language development**		
First words	11.5	15
Complete a sentence	18.5	24
**T** **oilet training**	Norms of age (month) of acquiring milestone by sex**	
	50th percentile	75th percentile
Toilet trained bladder – daytime	Girls: 32.5 Boys: 35	Girls: 36 Boys:39
Toilet trained bladder – nighttime	Girls: 34 Boys:36	Girls: 37 Boys:42
Toilet trained bowel (day + night)	Girls:31.5 Boys:34.5	Girls: 35 Boys:39

*According to The Denver II Developmental Screening Test (1992) [25]

**According to Schum, et al. (2002) [26]

Table. 7

**Table 7:** Correlation Matrix of intra-correlations between tics and comorbid psychopathology

	Tics (YGTSS)					OCD (CY-BOCS)			ADHD (SNAP)	ODD (SNAP)	Total difficulties score (SDQ)	Autism (ASSQ)
	Motor tics	Vocal tics	Total tic severity	Impairment	Yale global tic severity	Obsessions	Compulsions	Total OCD severity				
Tics (YGTSS)												
Motor tics	–	0.49***	0.84***	0.55***	0.75***	0.16***	0.21***	0.19***	0.21***	0.15**	0.21***	0.18***
Vocal tics		–	0.89***	0.52***	0.73***	0.22***	0.24***	0.24***	0.23***	0.19***	0.31***	0.25***
Total tic severity^+^			–	0.60***	0.86***	0.22***	0.26***	0.25***	0.25***	0.21***	0.31***	0.26***
Impairment				–	0.92***	0.21***	0.23***	0.24***	0.24***	0.16**	0.28***	0.21***
Yale global tic severity					–	0.23***	0.27***	0.26***	0.27***	0.19***	0.32***	0.26***
OCD (CY-BOCS)												
Obsessions						–	0.67***	0.87***	0.17***	0.19***	0.28***	0.05
Compulsions							–	0.93***	0.27***	0.25***	0.29***	0.21***
Total OCD severity								–	0.25***	0.25***	0.32***	0.16**
ADHD (SNAP)									–	0.66***	0.72***	0.56***
ODD (SNAP)										–	0.64***	0.45***
Total difficulties score (SDQ)^+^											–	0.57***
Autism (ASSQ)												–

**p* < 0.05, ***p* < 0.01

*YGTSS*, Yale Global Tic Severity Scale; *OCD*, Obsessive Compulsive Disorder; *CYBOCS*, Children’s Yale-Brown Obsessive Compulsive Scale; *ADHD*, Attention Deficit Hyperactive Disorder; *SNAP*, Swanson, Nolan and Pelham; *ODD*, Oppositional Defiant Disorder; *SDQ*, Strength and Difficulties Questionnaire; *ASSQ*, Autism Spectrum Screening Questionnaire.
